# When proprioceptive feedback enhances visual perception of self-body movement: rehabilitation perspectives

**DOI:** 10.3389/fnhum.2023.1144033

**Published:** 2023-05-12

**Authors:** Raphaëlle Schlienger, Claire De Giovanni, Michel Guerraz, Anne Kavounoudias

**Affiliations:** ^1^Aix-Marseille Université, CNRS, Laboratoire de Neurosciences Cognitives (LNC – UMR 7291), Marseille, France; ^2^Université Grenoble Alpes, Université Savoie Mont Blanc, CNRS, Laboratoire de Psychologie et NeuroCognition (LPNC – UMR 5105), Grenoble, France

**Keywords:** muscle tendon vibration, leg movement, proprio-visual integration, mirror therapy, kinesthesia

## Abstract

**Introduction:**

Rehabilitation approaches take advantage of vision’s important role in kinesthesia, using the mirror paradigm as a means to reduce phantom limb pain or to promote recovery from hemiparesis. Notably, it is currently applied to provide a visual reafferentation of the missing limb to relieve amputees’ pain. However, the efficiency of this method is still debated, possibly due to the absence of concomitant coherent proprioceptive feedback. We know that combining congruent visuo-proprioceptive signals at the hand level enhances movement perception in healthy people. However, much less is known about lower limbs, for which actions are far less visually controlled in everyday life than upper limbs. Therefore, the present study aimed to explore, with the mirror paradigm, the benefit of combined visuo-proprioceptive feedback from the lower limbs of healthy participants.

**Methods:**

We compared the movement illusions driven by visual or proprioceptive afferents and tested the extent to which adding proprioceptive input to the visual reflection of the leg improved the resulting movement illusion. To this end, 23 healthy adults were exposed to mirror or proprioceptive stimulation and concomitant visuo-proprioceptive stimulation. In the visual conditions, participants were asked to voluntarily move their left leg in extension and look at its reflection in the mirror. In the proprioceptive conditions, a mechanical vibration was applied to the hamstring muscle of the leg hidden behind the mirror to simulate an extension of the leg, either exclusively or concomitantly, to the visual reflection of the leg in the mirror.

**Results:**

(i) Visual stimulation evoked leg movement illusions but with a lower velocity than the actual movement reflection on the mirror; (ii) proprioceptive stimulation alone provided more salient illusions than the mirror illusion; and (iii) adding a congruent proprioceptive stimulation improved the saliency, amplitude, and velocity of the illusion.

**Conclusion:**

The present findings confirm that visuo-proprioceptive integration occurs efficiently when the mirror paradigm is coupled with mechanical vibration at the lower limbs, thus providing promising new perspectives for rehabilitation.

## Highlights

- The mirror paradigm evokes leg movement illusion but with a lower velocity than the actual movement seen.
- Adding proprioceptive feedback improves the clarity, amplitude, and velocity of the illusion.
- Combining visuo-proprioceptive feedback has potential in rehabilitation.

## 1. Introduction

Vision plays an essential role in the perception of self-body movements, as initially demonstrated by the vection phenomenon, i.e., the illusion of self-body movement evoked by a simple visual scene moving in front of a static participant (Brandt et al., 1972; Held et al., 1975; Guerraz and Bronstein, 2008; Blanchard et al., 2013; Kaneko et al., 2015; Harquel et al., 2020). For example, moving the background under a participant’s stationary hand may result in an illusory sensation of the hand rotating in the opposite direction of the actual visual scene, with a speed proportional to the rotation speed of the background (Blanchard et al., 2013). The mirror paradigm has also been used to study the involvement of vision in kinesthesia. The principle is to place a mirror parallel to the midline of a participant’s body, so the mirror reflects one limb while hiding the other. In healthy participants, the reflection of the hand in the mirror induces errors in reaching movements when there is a mismatch between the location of the reflected hand and that of the real hidden hand, the latter being perceived as located where the hand appears in the mirror (Holmes et al., 2004). The reflection of the moving hand in the mirror also produces an illusion of symmetrical bimanual movement, i.e., a kinesthetic illusion (Dohle et al., 2009; Guerraz et al., 2012; Metral et al., 2015).

The mirror paradigm is also a rehabilitation approach called “mirror therapy” currently used to relieve phantom limb pain in amputees (Ramachandran et al., 1995; Ramachandran and Altschuler, 2009) or to promote recovery from hemiparesis (Rosén and Lundborg, 2005; Dohle et al., 2009; Guerraz, 2015; Broderick et al., 2018). In the case of amputees, the mirror allows the image of the healthy limb to be seen instead of the residual limb. It is thought to strengthen the former representation of the missing limb by providing the brain with visual information, thereby counteracting the adverse effects of cerebral deafferentation related to the loss of sensory feedback from the amputated limb. However, the beneficial impact of mirror therapy is still debated and seems limited to a small proportion of patients (Giummarra and Moseley, 2011; Rothgangel et al., 2011; Guerraz, 2015; Barbin et al., 2016; Aternali and Katz, 2019; Guémann et al., 2023).

However, the movement of a body segment is multisensory in nature and involves the central nervous system combining and integrating all available signals to provide the most robust perception possible (Ernst and Bülthoff, 2004; Ehrsson and Chancel, 2019). The mirror illusion, often considered as a prototypical visual illusion, is no exception to this rule and can only be understood when considered in its multisensory (and motor) context. In the mirror paradigm, the mirror feedback is accompanied by a physical active or passive displacement of the contralateral body segment. However, more importantly, it conflicts with proprioceptive afferents of the hidden segment, the latter informing the central nervous system that it is not actually moving, which limits the potential of visual manipulation (Dupraz et al., 2022). The crucial role of proprioceptive and visual integration is well-revealed using the mirror box, where participants place their hands into a box separated by a mirror and tap with both hands while viewing one reflected hand in the mirror. The congruency between visual and proprioceptive information determines the experience of the participants. If both hands are moving synchronously, the participants will feel a shift in the perceived location of their hidden hand toward the reflected one, as well as an illusion of ownership where they perceive the reflected hand as their own. However, this illusion is reduced when the two hands tap out of phase, indicating an incongruence between visual and proprioceptive information (Medina et al., 2015; Wittkopf et al., 2017). The use of the mirror therapy for amputees has been largely inspired by the work of Ramachandran (Ramachandran et al., 1995; Ramachandran and Altschuler, 2009) and the principle of reafference (von Holst and Mittelstaedt, 1950) and aims to re-establish the congruency between efferent commands and the afferent signals. The mirror feedback fulfills this function, but only partially, given the presence of proprioceptive signals still conveying signals from a static body segment (or from a stump in amputees), conflicting with both the motor command and visual feedback. Therefore, providing the brain with sensory feedback from the missing limb limited to the visual domain may not be sufficient to drive appropriate central reorganization within the entire sensorimotor network. Notably, it has been reported that the combination of visual signals and active movements of the healthy limb is more effective than passive vision alone (Lotze et al., 1999; Ortiz-Catalan et al., 2014).

By contrast, hand movement illusions elicited by wrist muscle tendon vibrations in healthy adults have been associated with activations similar to those observed during real hand movements, including the contralateral primary sensorimotor cortex, but also the bilateral supplementary motor area, posterior parietal lobules, basal ganglia, insula, and ipsilateral cerebellum (Naito et al., 1999; Casini et al., 2006; Duclos et al., 2007; Kavounoudias et al., 2008; Cignetti et al., 2014; Landelle et al., 2020).

In addition, perceiving self-body movements is enhanced by congruent multisensory inputs from the limb, particularly visual combined with proprioceptive cues (Guerraz et al., 2012; Blanchard et al., 2013; Bisio et al., 2019). Regarding the upper limbs, Guerraz et al. (2012) showed in healthy adults that the mirror illusion experience was enhanced when a congruent vibration was simultaneously applied on the hidden resting arm behind the mirror, which has been recently confirmed when the hidden arm is replaced by a visual avatar in virtual reality (Dupraz et al., 2022).

However, much less is known about the perceptual benefits of combining vision and proprioceptive cues at the lower limbs, and results found at the upper limbs may not be generalized to the lower limbs. Indeed, we know that proprioceptive performance varies based on the body site, as proprioceptive scores at different joint levels did not significantly correlate (Waddington and Adams, 1999; Han et al., 2013). Additionally, lower limb movements are typically less visually controlled than upper limb movements in everyday life, suggesting that the beneficial impact of combining visual and proprioceptive inputs may differ between the upper and lower limbs. Nevertheless, a recent microneurographic study has suggested that visual feedback can modulate muscle spindle sensitivity from leg muscles, indicating that interactions between visual and proprioceptive inputs can occur at the lower limbs (Ackerley et al., 2019). Mirror therapy, which has been tested in amputees (Seidel et al., 2011; Ramadugu et al., 2017; Rothgangel et al., 2018) and stroke patients (Broderick et al., 2018; Cui et al., 2022) to alleviate phantom limb pain or improve motor outputs, has also shown promising results at the lower limbs, although its systematic benefit remains controversial at both upper and lower limbs (Barbin et al., 2016; Aternali and Katz, 2019; Guémann et al., 2023).

Therefore, the present study aimed to explore the benefit of combined visuo-proprioceptive feedback from the lower limbs of healthy participants. For this purpose, we compared the amplitude and velocity, as well as the saliency and the relative speed of right leg movement illusions induced in 23 healthy young volunteers under three stimulation conditions: (i) only visual, i.e., the mirror paradigm, (ii) only muscle proprioceptive, i.e., the mechanical vibration applied on hamstring muscle tendons, and (iii) bisensory, i.e., the congruent combination of visual and proprioceptive stimulation. The clinical perspectives of the present findings are discussed.

## 2. Materials and methods

### 2.1. Participants

A total of 23 healthy young adults (15 women and eight men, mean age = 23.4 ± 2.4 years, all were university students, at the bachelor or master level) underwent a familiarization test. None of them had any history of neurological or sensorimotor diseases, nor were they receiving medical treatment. All the participants were recruited through an advertisement posted in our university and gave written, informed consent and the study was approved by the local ethics committee (CCPP Marseille Sud 1 #RCB 2010- A00359-30) in accordance with the Declaration of Helsinki.

The sample size of the present study was predicted by a power analysis (G*Power analysis software version 3.1.9.6) based on a previous independent study published in Guerraz et al. (2012) using the same kinds of visuo-proprioceptive stimulation but applied at the upper limbs. The prediction of the sample size required to reach 95% statistical power in a mean comparisons of paired data set at a significance level of 0.05 was 19 participants.

As the goal of the present study was to investigate visuo-proprioceptive interactions, four participants were not included in the complete experiment since they did not feel any illusion in the visual (three participants) or proprioceptive (one participant) conditions.

### 2.2. Stimulations

#### 2.2.1. Visual stimulation (V)

**FIGURE 1:**
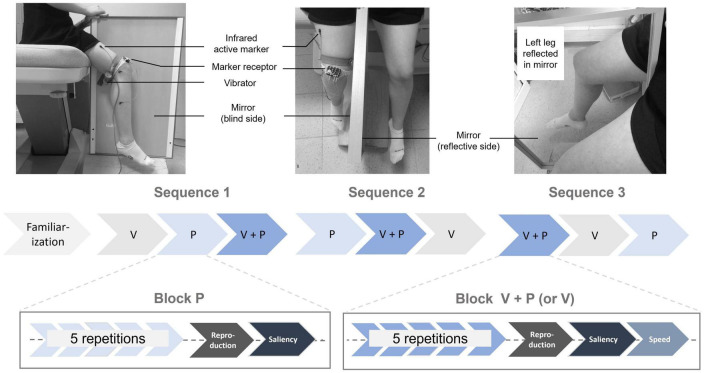
Experimental setup: The participant sat with a mirror between their legs and was asked to look at the reflection of their left leg in the mirror. The physical right leg remained static and out of sight. A mechanical vibrator was attached to the hamstring muscle tendon of the hidden right leg, along with three sensors to capture its movements during the reproduction task. In the V and V+P conditions, the participant actively moved their left leg in extension, while in the P and V+P conditions, the hamstring muscle was stimulated for 10 s. Experimental protocol: After a familiarization phase, participants underwent a set of three stimulation sequences, each including three blocks of 5 repetitions of the three stimulation conditions (V: visual, P: proprioceptive, V+P = visuo-proprioceptive). After each stimulation block, they reproduced the largest of the perceived extension illusions with their right leg.

A mirror (80 cm long and 50 cm wide) was placed between the participant’s two legs so that they could see only the reflection of their left leg and not the hidden right leg (vibrators “vibrasens” developed by Technoconcept company, Manosque, France). On each trial, the participant was asked to voluntarily extend their left leg during a 10 s interval defined by two beeps. The participant was told to look at the reflection of the moving left leg in the mirror, resulting in an illusion of right leg extension, though this right leg remained motionless and hidden behind the mirror (Figure 1).

#### 2.2.2. Proprioceptive stimulation through tendon vibration (P)

The proprioceptive stimulation consisted of a mechanical vibration using a vibrator (length: 8 cm, diameter: 3 cm) attached by an elastic band to the hamstring tendon of the right leg, which remained static (Figure 1). The vibration was delivered at low amplitude (0.5 mm peak to peak) and constant frequency (60 Hz) for 10 s. With eyes closed, in a relaxed position, the participant was thus able to perceive an extension illusion of their right leg, i.e., a movement corresponding to the stretch of the vibrated muscle.

We know that primary muscle spindle afferents are sensitive to mechanical vibration and respond in a one-to-one mode for vibration frequencies ranging between 20 and 80 Hz (Roll and Vedel, 1982). By increasing the vibration frequency from 20 to 80 Hz, the velocity of the resulting illusion increases proportionally (Blanchard et al., 2013). As the main goal of the study was to investigate the effect of a combination of visual-proprioceptive stimulation on leg movement, we chose a vibration frequency of 60 Hz that did not evoke an illusion of maximum velocity to allow us to observe the improvement associated with the addition of the visual stimulus.

#### 2.2.3. Combination of visual and proprioceptive stimulation (V+P)

A hamstring tendon vibration of the right leg (time = 10 s, frequency = 60 Hz) was applied while the participant simultaneously extended the left leg. As in the visual condition, the participant had to look at the reflection of their left leg in the mirror during the 10 s-time interval marked by two beeps.

Beep signals and vibration delivery were performed using a National Instruments card (NI PCI-6229) controlled using a software program implemented in a Labview 8.2 environment.

### 2.3. Experimental protocol

The participants were asked to sit without their legs touching the floor. There were three experimental stimulation conditions: visual (V), proprioceptive (P), and visuo-proprioceptive (V+P). The experiment was conducted in two steps: (i) A familiarization period to select only participants able to experience the visual and proprioceptive illusions and (ii) three test sequences, including the three stimulation conditions delivered in blocks of five repetitions. The order in which the three stimulation blocks were presented within a sequence was randomized, as was the sequence order across the participants. The entire experiment lasted about 60 min (Figure 1).

### 2.4. Data acquisition and analysis

#### 2.4.1. Illusion saliency

After each run, the participant orally reported illusion saliency on a subjective scale ranging from 0: “no illusion” to 4: “clear sensation of movement.” Because the salience index was an ordinal and not a continuous value, the difference in saliency rating by condition was analyzed with a Friedman non-parametric statistical test (α = 0.05) followed by *post-hoc* tests (paired Wilcoxon tests, Bonferroni-corrected *p*-values).

#### 2.4.2. Movement illusion reproduction

After five repetitions of a stimulation condition (V, P or V+P), the participant was asked to reproduce the largest movement illusion felt in their hidden right leg. This movement was recorded by a motion capture system (CODAmotion, Charnwood Dynamics, UK) consisting of a camera and three active infrared markers. The markers were placed on the lateral side of the right knee joint, at the thigh, kneecap, and tibia levels, to capture angular deviations of the knee during the participant’s reproduction. The camera recorded each marker’s position in space (3D) with a 10 Hz frequency, and the angular deviations of the knee were extracted with CODAmotion Analysis software (version 6.78.2). The maximal angular deviation of the knee joint angle at rest thus represented the amplitude of the extension reproduced by the participant for each block of each sequence. The mean amplitudes of reproduced illusions in the three conditions were then compared with a one-way repeated measures ANOVA (three levels: V, P and V+P) followed by *post-hoc* tests (paired Student *t*-tests, with Bonferroni-corrected *p*-values) after normal distribution verification (Shapiro-Wilk test, *p* = 0.28). Because the data were not spherical (Mauchly test: *p* = 0.0003), the *p*-value obtained through ANOVA was corrected with the Greenhouse-Geisser correction. The threshold for statistical significance was set at *p* < 0.05. The mean velocity of the illusion (°/s) was also calculated from the onset of the illusion up to the maximum angular deviation as measured with the capture motion recordings using the least square method to obtain a linear regression of the data. Because the velocity data did not satisfy the normality criteria, we tested the differences between the three stimulation conditions using non-parametric statistical tests: a Friedman test followed by Wilcoxon’s paired tests.

#### 2.4.3. Relative speed perception

For conditions including the mirror (V and V+P), the participant was also asked to estimate the relative speed of the perceived illusion on a subjective scale ranging from 0: “no illusion,” to 10: “Illusion sensation as fast as the moving leg seen in the mirror,” to 20: “Illusion sensation twice as fast as the observed movement.” This subjective estimation, inspired by Guerraz et al. (2012), will allow a comparison of present results observed in the lower limb to those previously reported in the upper limb. Because these were ordinal and not continuous values, the estimated illusion speeds between the V and V+P conditions were compared by a Wilcoxon non-parametric statistical test (α = 0.05).

All analyses were performed with python toolboxes SciPy (v1.10.0) (Virtanen et al., 2020), Pingouin (v0.5.10) (Vallat, 2018), and statsmodels (v0.10.2) (Seabold and Perktold, 2010).

## 3. Results

### 3.1. Saliency of movement illusions

**FIGURE 2:**
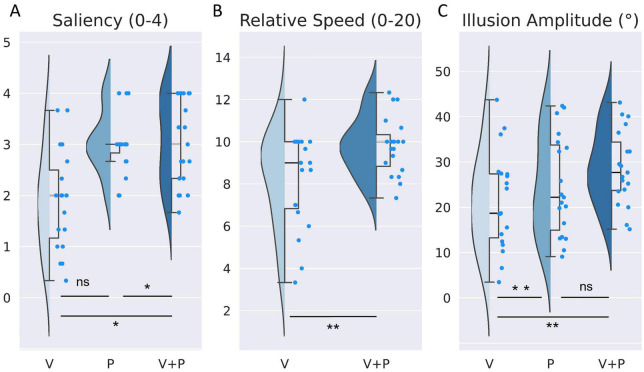
Comparison of the effects evoked by visual (V), proprioceptive (P), and visuo-proprioceptive (V+P) stimulation on **(A)** saliency of illusions (ranging from 0 = no illusion, to 4 = clear sensation of movement), **(B)** perceived vs. actual speed of the leg in the mirror (from 0: “absence of illusion”, to 10: “Illusion sensation as fast as moving leg seen in the mirror”, to 20: “Illusion sensation twice as fast as the observed movement”) and **(C)** illusion amplitude (extracted from the reproduction task), Half violin plots show distribution of indices, and Half box plots show medians, first and third quartiles (upper and lower box hinges) and maximum and minimum values (whiskers). Bright blue points represent each participant’s raw individual data. *p < 0.05, **p < 0.01, ns, not significant.

After each stimulation, participants reported the illusion saliency according to a subjective scale ranging from 0 (“no illusion”) to 4 (“clear sensation of movement”) (Figure 2A).

A Friedman non-parametric analysis showed a significant effect of stimulation condition (*Q* = 13.77; *p* = 0.001). More precisely, the saliency of the illusion during a combined visuo-proprioceptive (V+P) stimulation was significantly higher than that reported during the visual stimulation alone (V) (*W* = 12.5; *p* = 0.004) but not during the proprioceptive (P) stimulation alone (*W* = 58; *p* = 1) (Table 1). In addition, the proprioceptive stimulation gave rise to higher salient illusions of movement than the visual stimulation (*W* = 7.0; *p* = 0.003).

**TABLE 1:** Comparison of the saliency, relative speed, amplitude, and velocity of movement illusions of all the participants (*n* = 19) according to the stimulation conditions (V, P, and V+P).

		Indices	Statistics			
		**Mean ± SD**	**Main effect**	**V vs. P**	**V vs. V+P**	**P vs. V+P**
Saliency (0–4)	V	1.91 ± 1.0	Friedman *Q* = 13.77 *p* = 0.001***	*W* = 7.0 *p*_*corr*_ = 0.003**	*W* = 12.5 *p*_*corr*_ = 0.004**	*W* = 58.0 *p*_*corr*_ = 1.000
	P	3.04 ± 0.6				
	V+P	3.03 ± 0.8				
Relative speed (0–20)	P	8.17 ± 2.3	–	–	–	*W* = 26.5 *p* = 0.010**
	V+P	9.80 ± 1.4				
Illusion amplitude (°)	V	21.37 ± 10.8	ANOVA *F*_(2, 36)_ = 5.12 *p* = 0.027*	*t* = −1.09 *p*_*corr*_ = 0.870	*t* = −3.31 *p*_*corr*_ = 0.012*	*t* = −2.96 *p*_*corr*_ = 0.025*
	P	24.67 ± 11.1				
	V+P	28.76 ± 8.2				
Illusion velocity (°/s)	V	5.04 ± 3.2	Friedman *Q* = 8.11 *p* = 0.017*	*W* = 40.0 *p*_*corr*_ = 0.145	*W* = 24.0 *p*_*corr*_ = 0.017*	*W* = 65.0 *p*_*corr*_ = 1.000
	P	7.42 ± 5.8				
	V+P	6.79 ± 3.8				

The significance threshold of the statistical tests was set at 0.05 and *post-hoc* tests were corrected for multiple comparisons when necessary.

*p < 0.05, **p < 0.01, and ***p < 0.001.

### 3.2. Relative speed perceived in the right leg with respect to the actual velocity of the left leg reflected in the mirror

In both conditions with mirror (V and V+P), participants reported the relative speed of perceived illusion in their motionless right leg with respect to the actual velocity of the left leg, on a subjective scale ranging between 0 and 20 (0: no illusion, 10: equal speed, 20: speed of the illusion twice as high as that of the actual moving leg seen in the mirror). On average, participants perceived the movement illusion as slower than the actual movement of the leg reflected in the mirror with a mean score of 8.17, which could roughly correspond to a movement 20% slower than the actual movement. Adding a proprioceptive stimulation to the visual one significantly increased the relative speed of the illusion (*W* = 26.5; *p* < 0.01), with an illusory speed approaching the actual leg velocity and a mean score of 9.8, close to the equality score of 10 (Figure 2B and Table 1).

At the individual scale, fourteen of the nineteen participants rated the speed of the illusion faster in the V+P condition compared to the V condition, one participant reported no difference, and only four rated the speed lower than that of the actual moving leg.

### 3.3. Amplitude and velocity of movement illusions reproduced in the three stimulation conditions

As shown in Figure 2C, the maximal amplitude extracted from the angular deviation of the knee (based on the reproduction of the illusion with the right leg) differed significantly according to stimulation conditions [ANOVA main effect F(2,36) = 5.12; *p* < 0.05, Table 1]. *Post-hoc* analysis revealed that the illusion amplitude was significantly higher in the bimodal V+P condition compared to the two unimodal V or P conditions (*t* = –3.31 for V vs. V+P, and *t* = –2.96 for P vs. V+P, *p* < 0.05, Table 1). In contrast, no difference was observed between the two unimodal conditions V and P (*t* = 1.09; *p* = 0.87). The velocity of the illusions followed the tendency of the maximal amplitude index. The velocity of visual illusions was also found significantly smaller than that evoked in the bimodal condition, while the velocity of the proprioceptive illusions did not significantly differ from those induced in the bimodal condition. However, contrary to the amplitude value, the velocity of the proprioceptive illusions did not reach a significant difference with the velocity of the visual illusions (*W* = 40; *p* = 0.145) (Table 1).

## 4. Discussion

The present study provides new results related to the possibility of evoking mirror illusions in the lower limb level and enhancing this perception by simultaneously providing visual and proprioceptive stimulation. To date, this phenomenon has been reported almost exclusively in the upper limb, although it may open important perspectives for rehabilitation purposes.

### 4.1. The kinesthetic benefit of adding proprioceptive feedback in the mirror paradigm

Among the 23 participants initially tested, only three were unable to experience a leg movement illusion when looking at their right moving leg in the mirror, and one when vibrating the hamstring muscle of their left leg. Among the remaining 19 participants included in the experiment, only 10% of the trials did not evoke any mirror illusion, which is consistent with the illusion occurrence usually reported in the literature (Metral et al., 2015; Chancel et al., 2016b,^2017^). We also observed that the mirror paradigm evoked leg movement illusion in the visual condition (V) but with a lower relative speed than the actually seen movement. This finding is consistent with mirror illusions evoked in the upper limb (Guerraz et al., 2012) but also with vection illusions evoked by a rotating visual or tactile background under participants’ hand (Blanchard et al., 2013; Chancel et al., 2016a). The general explanation is that the movement information provided by the visual stimulation conflicts with the proprioceptive and tactile feedback from the static limb, resulting in a reduced illusion of movement. Indeed, it has been shown that masking proprioceptive information by co-vibration applied to the antagonist arm muscles (Biceps and Triceps of the arm subjected to the illusion) can increase the velocity of the mirror arm illusion and decrease its latency (Guerraz et al., 2012; Dupraz et al., 2022). The same effects of proprioceptive masking have been reported in the case of visual or tactile hand vection (Chancel et al., 2016a).

Consistently, we found that adding a proprioceptive signal congruent to the visual feedback increased the saliency, amplitude, and relative speed of the mirror illusion. In the bimodal condition, the participants perceived a movement illusion as fast as the moving leg reflected in the mirror. In addition, the amplitude of the leg illusion increased on average by 35 and 15% during the visuo-proprioceptive stimulation compared to separate visual or proprioceptive stimulation, respectively. In the arm, Guerraz et al. (2012) reported a similar effect with an increase in illusion velocity of 65 and 17% in the bimodal stimulation compared to the isolated visual and proprioceptive conditions.

Interestingly, although illusion amplitude for both unimodal stimulations did not differ significantly, participants perceived a significantly clearer movement illusion in the proprioceptive (P) than in the visual (V) condition. One reason may be that the mirror-induced visual illusion activates mainly visual areas (Matthys et al., 2009) and not the primary sensorimotor cortex. This is also the case during a movement illusion evoked by watching a video of one’s own hand moving. Kaneko et al. (2015) showed that participants can experience a hand movement illusion while watching a pre-recorded video of their own hand moving (Self Hand) but not that of someone else’s hand. By contrasting the two conditions (self vs. other hand), they found brain activations in the premotor cortex, parietal regions, as well as in the insula and putamen, but not in the primary motor and somatosensory cortices. This finding aligns with previous research indicating that watching a video of a finger movement only increases excitability in M1 when paired with a congruent proprioceptive vibration, which induces an illusion of the finger movement in the same direction as that seen in the video (Bisio et al., 2019). Indeed, movement illusions elicited by vibrating muscle tendons in healthy adults have been associated with activations similar to those observed during actual hand movements, including the contralateral primary sensorimotor cortex, but also the bilateral supplementary motor area, posterior parietal lobules, basal ganglia, insula, and ipsilateral cerebellum (Naito et al., 1999; Kavounoudias et al., 2008; Cignetti et al., 2014; Landelle et al., 2020). The finding that muscle tendon vibration increases M1 excitability while superficial tactile stimulation or visual observation of an action is not sufficient to induce M1 plasticity has been suggested to be due to the role of conscious perception of movement. This was shown in a study by Bisio et al. (2019), who used transcranial magnetic stimulation and found that the level of plasticity induced in M1 varied positively with the vividness of the proprioceptive illusions. These results suggest that the conscious perception of movement plays a crucial role in modulating M1 excitability, and that the vividness of proprioceptive illusions may be an important factor in determining the level of plasticity induced.

The present results confirm that muscle proprioceptive and visual signals are integrated by the CNS and extend what has been previously described for the upper limb (Guerraz et al., 2012) to the lower limb, despite the fact that lower limb displacements are much less visually controlled in daily life than arm actions and definitely appear much less in the individual’s visual field. However, it must be acknowledged that the role of visual cues in the present mirror paradigm may be slightly overestimated. Indeed, the movement of the leg in the mirror is accompanied by an active displacement of the contralateral leg, i.e., it is accompanied by a motor command to move it and by muscle proprioceptive signals related to that contralateral leg. Although it is not clear whether a motor command toward a segment can affect perception of the contralateral segment, it has been demonstrated in the mirror paradigm (Chancel et al., 2016b,^2017^) and in its adaptation in virtual reality (Giroux et al., 2018) that contralateral muscle proprioceptive signals impact motion perception. For instance, masking muscle proprioceptive afferents of the arm reflected in the mirror (by synchronous co-vibration of antagonist muscles) decreases the strength of the illusory movement of the arm hidden behind the mirror (Chancel et al., 2016b). Therefore, the mirror illusion is not purely visual in origin but results from the combination of visuo-proprioceptive signals from both arms, which likely applies to the legs.

### 4.2. Clinical perspectives

The present findings may have a great impact on clinical perspectives. Using a similar mechanical vibration to stimulate proprioceptive afferents but set at 80 Hz instead of 60 Hz, Roll et al. (2012) found that illusory movement sensations induced during temporary hand immobilization could prevent disruption of the sensorimotor network usually associated with arm disuse in healthy adults, including the contralateral primary sensorimotor region but also the dorsal premotor cortex, the inferior parietal lobule and the supplementary motor area. Avanzino et al. (2014) also demonstrated that a proprioceptive treatment using muscle vibration not only prevented a decrease in contralateral M1 excitability after 1 day of a unilateral hand immobilization but also prevented hemispheric imbalance between the two M1 cortices. In contrast, a pure tactile treatment did not show the same benefits.

Furthermore, movements of the phantom limb and pain seem to be closely related in amputees: Gagné et al. (2009) reported that amputees who can voluntarily move their phantom limb have less pain and present a reduced central reorganization, compared to amputees who have less “mobile” phantoms. Interestingly, voluntary movements of the phantoms trigger peripheral contractions of the stump muscles (Reilly et al., 2006) that might, in turn, stimulate muscle spindle afferents from that stump. Similar contractions have never been reported in the amputated limb during mirror therapy.

However, a limitation of the present study is that participants who did not experience any movement illusion under visual or proprioceptive stimulation were not included in the experiment. Although this only involved 4 out of 23 participants, it limits the generalizability of the present results by restricting it to the population sensitive to kinesthetic illusion. In addition, we were dealing with a young population (20–29 years of age), whereas the clinical population suffering from sensorimotor deficits, in particular amputees, especially those of vascular origin, is most often older. In addition, after 65 years old, a greater functional deficit in muscle proprioception than in vision was reported in healthy adults (Chancel et al., 2018). Therefore, further experiments should be conducted on a wider age range in order to refine personalized rehabilitation protocols.

All in all, one can hypothesize that providing the brain with only visual feedback relative to a missing or impaired limb may not be optimal to drive the appropriate central reorganization in the whole sensorimotor network. This would explain why mirror therapy, despite being often considered as a promising tool, seems to have a limited impact on amputees (Rothgangel et al., 2011; Guerraz, 2015) and stroke patients (Dohle et al., 2009; Rothgangel et al., 2011). The present study shows that providing the brain with a combination of proprioceptive and visual signals simulating limb movements results in a greater movement perception, which may have a greater therapeutic impact on phantom pain issues. Moreover, in the mirror paradigm, proprioceptive stimulation alone leads to a clearer sensation than visual stimulation, which may also provide an alternative to mirror therapy for amputees who experience adverse reactions when suddenly confronted with their missing limb. More generally, the present study highlights the importance of considering muscle proprioception in combination with other senses to promote recovery of kinesthetic functions in patients with sensorimotor disabilities.

## Data availability statement

The raw data supporting the conclusions of this article will be made available by the authors, without undue reservation.

## Ethics statement

The studies involving human participants were reviewed and approved by Comité de Protection des Personnes Sud Méditerranée I. The patients/participants provided their written informed consent to participate in this study.

## Author contributions

AK and MG contributed to the conception and design of the study. CD and AK performed the experiment. RS, CD, and AK performed the data analysis and statistics. RS and AK wrote the first draft of the manuscript. MG wrote sections of the manuscript. CD assisted with the figures. All authors contributed to the revision of the manuscript, read, and approved the submitted version.
